# Geographically weighted principal component analysis for characterising the spatial heterogeneity and connectivity of soil heavy metals in Kumasi, Ghana

**DOI:** 10.1016/j.heliyon.2021.e08039

**Published:** 2021-09-22

**Authors:** Eric N. Aidoo, Simon K. Appiah, Gaston E. Awashie, Alexander Boateng, Godfred Darko

**Affiliations:** a Department of Statistics & Actuarial Science, College of Science, Kwame Nkrumah University of Science and Technology, Kumasi, Ghana; b Department of Mathematics, College of Science, Kwame Nkrumah University of Science and Technology, Kumasi, Ghana; c Department of Chemistry, College of Science, Kwame Nkrumah University of Science and Technology, Kumasi, Ghana

**Keywords:** Soil pollution, Heavy metals, Principal component analysis, Spatial heterogeneity, Geographically weighted principal component analysis

## Abstract

The use of principal component analysis (PCA) for soil heavy metals characterization provides useful information for decision making and policies regarding the potential sources of soil contamination. However, the concentration of heavy metal pollutants is spatially heterogeneous. Accounting for such spatial heterogeneity in soil heavy metal pollutants will improve our understanding with respect to the distribution of the most influential soil heavy metal pollutants. In this study, geographically weighted principal component analysis (GWPCA) was used to describe the spatial heterogeneity and connectivity of soil heavy metals in Kumasi, Ghana. The results from the conventional PCA revealed that three principal components cumulatively accounted for 86% of the total variation in the soil heavy metals in the study area. These components were largely dominated by Fe and Zn. The results from the GWPCA showed that the soil heavy metals are spatially heterogeneous and that the use of PCA disregards this considerable variation. This spatial heterogeneity was confirmed by the spatial maps constructed from the geographically weighted correlations among the variables. After accounting for the spatial heterogeneity, the proportion of variance explained by the three geographically weighted principal components ranged between 85% and 89%. The first three identified GWPC were largely dominated by Fe, Zn and As, respectively. The location of the study area where these variables are dominated provides information for remediation.

## 1 Introduction

Assessment of soil heavy metal pollution has been given much attention for decades because of its consequences to human health and the environment (Kowalska et al. 2018; Lu et al. 2020; Wu et al. 2020). The consequences of this type of pollution may be beneficial or harmful to biological systems depending on the type of metals involved and the level (Caussy et al. 2003; Darko et al. 2017b). For instance, Darko et al. (2017b) argued that whilst metals such as copper, zinc and chromium are essential for biological functioning in humans, other metals such as mercury, cadmium and lead have no benefit to biological systems.

The sources of these metals in soils may be natural components or due to anthropogenic activities (Kähkönen et al. 1997; Bretzel and Calderisi 2006; Darko et al. 2017a; Fernández et al. 2018). However, heavy metal pollution in soils in urban areas is mostly influenced by human activities associated with transport and building as well as incineration of domestic and industrial waste (Bretzel and Calderisi 2006; Darko et al. 2017a). According to Bretzel and Calderisi (2006), the physical, chemical and biological properties of soils in urban areas have been modified by urbanization. This modification does not only degrade the quality of the soil, atmosphere plants’ and water bodies but could also pose a threat to human health if not monitored (Fernández et al. 2018). Thus, assessment of soil heavy metal pollution is of great importance for decision making and policy formulation.

Due to different sources of contamination, an assessment of soil heavy metal pollution may be difficult (Borůvka et al. 2005). In an attempt to assess diffuse pollution sources of soil heavy metals, different statistical methodologies have been implemented. One of the most commonly used approaches for this purpose is the principal component analysis (PCA) (Borůvka et al. 2005; Bretzel and Calderisi 2006; Chang et al. 2009; Reid and Spencer 2009; Lu et al. 2011). The PCA is a multivariate statistical technique used in reducing the dimensionality of correlated multivariate data. For instance, suppose there are several soil heavy metal pollutants measured at different locations in a study region. The PCA aims at transforming the measured correlated pollutants to a smaller set of uncorrelated variables, called principal components. The principal components are new set of uncorrelated variables which are constructed as a linear combination of the original correlated variables. These new variables represent the direction of the data that explains the major patterns of the variations in the original data.

The PCA has been widely applied for soil heavy metals pollution characterization and offers considerable benefits of understanding multiple sources of soil pollution (Reid and Spencer 2009; Lu et al. 2011). These benefits include identification of multivariate relationships and dependencies among soil heavy metal variables, and reduction of high-dimensionality of these variables. However, heavy metal pollutants are highly persistent and their concentration varies across contaminated site (Borůvka et al. 2005; Fernández et al. 2018). Thus, the application of the conventional PCA to characterize such variables may ignore the spatial heterogeneity in the data and this may be problematic (Harris et al. 2015). In addition, the covariance structure used in conventional PCA is assumed to be constant across the study area and this may bias its output in the presence of spatial heterogeneity (Charlton et al. 2010). Accounting for such spatial heterogeneity will improve our understanding with respect to the distribution of the most influential heavy metals in each identified principal components.

To account for spatial heterogeneity in soil heavy metals data, the geographically weighted principal component analysis (GWPCA) was used. The use of the GWPCA does provides additional information which is obscured in the conventional PCA (Kumar et al. 2012). For instance, remediation regarding soil pollution problems will require that the influential soil pollutants and their spatial location is known. Such information will influence the choice of the remediation method and policy formulation regarding area delineation. In addition, information regarding the dominant soil heavy metal in the area, will guide us to detect the sources and all these will improve our understanding on the problem and how to mitigate it. In this study, GWPCA was applied to characterise the spatial heterogeneity and connectivity of soil heavy metals in Kumasi Ghana. The spatial connectivity refers to the interaction of the variables at spatially distinct location. Such information is important to provide a better understanding of the variables that dominate in any given part of the study area and the spatial relationships between different soil heavy metals.

## 2 Materials and methods

### 2.1 Study area and data description

**Figure 1:**
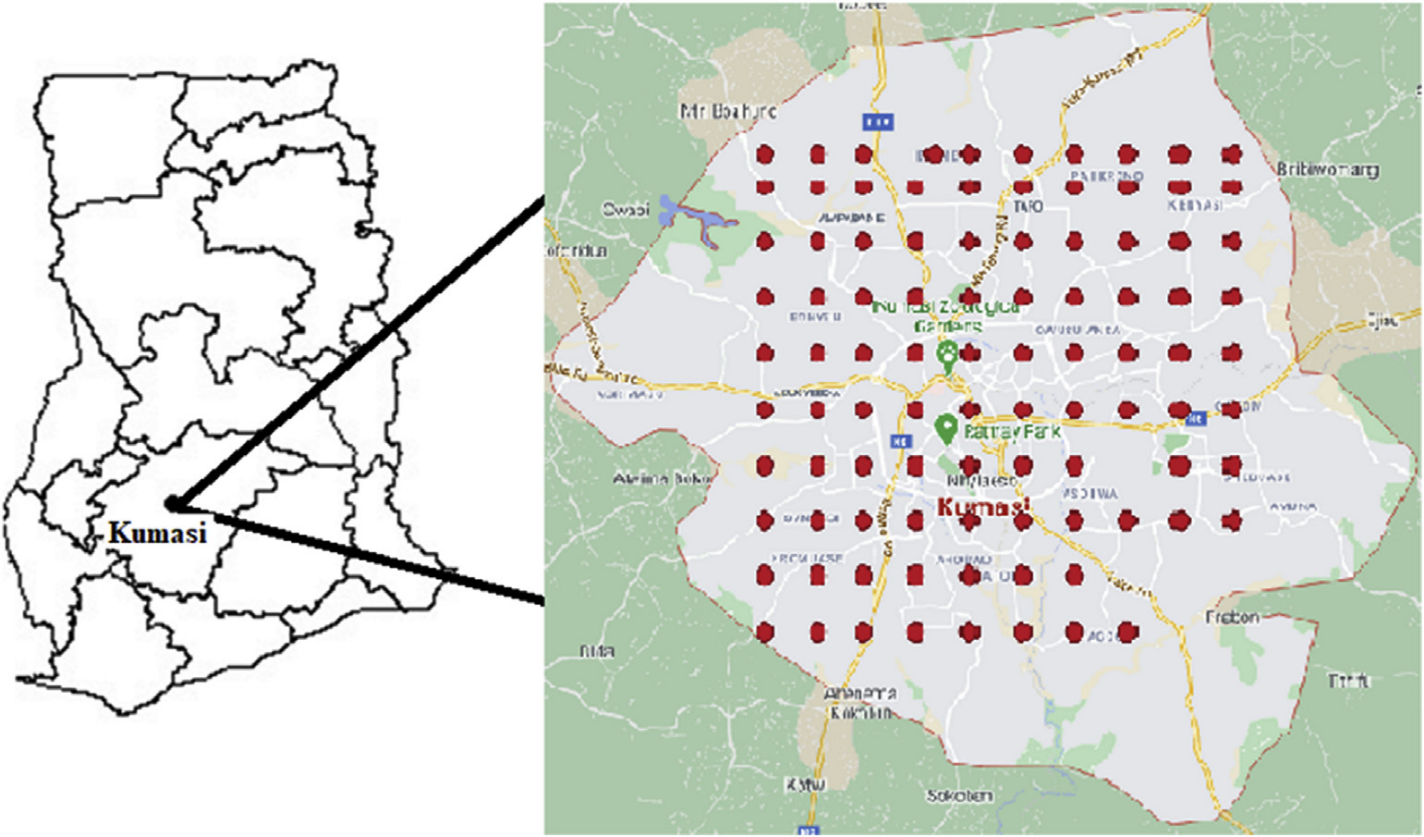
Map of Ghana showing the location of Kumasi and the sampling locations.

The study was limited to the Kumasi metropolis located between latitude 6.6^0^ North and longitude 1.6^0^ West of Ghana (Figure 1). The Kumasi metropolis is the second most populous city in Ghana with population of 3,206,000. The city of Kumasi serves as the regional capital of Ashanti Region of Ghana with total land area of 254 km^2^. The economic activities in the city are predominantly commerce, trade and industry (Darko et al. 2017a). The largest auto-mechanic workshops dealing with metal engineering and vehicle repairs are also located in the city of Kumasi (Iddrisu et al. 2002).

The soil heavy metals data used in this study were collected across the commercial hub of Kumasi by Darko et al. (2017a). The heavy metals were extracted from topsoil samples which were collected in areas where major commercial activities are concentrated in the Kumasi metropolis. The sample collection process span between November and December, 2015. The sampling locations were determined using a square grid of size 0.5 km by 0.5 km in areas where most of these commercial activities are concentrated. At each location, a trowel was used to collect the soil samples within a depth of 0–10 cm into a high-density polyethylene bag. The heavy metals concentration (mg/kg) were measured at 107 sampling locations in the study region of which 94 sampling locations were considered in this study (Figure 1). According to Darko et al. (2017a), each soil sample was air-dried and sieved through a <250 μm mesh sieve. It was then analysed for total metals by ICP-MS after modified aqua regia digestion. According to the authors, 0.5 g sample was added to 3 mL of 1:1:1 HCI–HNO_3_–H_2_O mixture. The mixture was digested at 95 °C for an hour in a heating block. The sample was made to volume with dilute HC1 and analysed by ICP-MS based on United States Environmental Protection Agency standard operating protocols and methods. Detailed information about the analysis is documented in Darko et al. (2017a). The variables (soil heavy metals) considered in this study includes As, Cr, Cu, Fe, Mn, Ni, Zn, and Cd.

### 2.2 Principal component analysis (PCA)

The conventional PCA is a multivariate statistical technique used for extracting information from higher dimensional correlated multivariate data by projecting it into a lower dimensional sub-space (Forkman et al. 2019). Given a set of correlated variables (such as soil heavy metals), a smaller set of uncorrelated variables called principal components (PCs) which are linear combinations of the original variables are produced. These new sets of variables are computed from the variance-covariance matrix of the original variables. The first few PCs accounts for most of the variations in the original data set. The concept and applications of PCA have been widely discussed in many literatures (Johnson and Wichern 2002; Abdi and Williams 2010). However, its extensive applications in various disciplines does not account for the presence of spatial heterogeneity in the data, thereby reducing the information that can be obtained from such data. Hence, the use of geographically weighted principal component analysis for an environmental based decision in an urban area becomes useful (Harris et al. 2015). The use of the GWPCA will provide additional information which is obscured in the conventional PCA (Kumar et al. 2012). Such information may include the influential soil pollutants and their spatial distribution within the study area.

### 2.3 Geographically weighted principal component analysis

The GWPCA which was introduced by Fotheringham et al. (2002) extends the conventional PCA by incorporating the geographical information of the variables in the calculation of the principal components (PCs). This type of computation allows us to account for the spatial heterogeneity which may be ignored by the conventional PCA. In the GWPCA, the PCs are computed from the variance-covariance matrix which are weighted as a function of the spatial distances. The geographically weighted variance-covariance matrix is expressed as:

(1) $$\sum \left(u_{i},\;v_{i}\right)=X^{\text{T}}W\left(u_{i},\;v_{i}\right)X$$

where **X** is an matrix of the original variables (soil heavy metals) with *n* representing the number of observations and *p* represents the number of individual variables. The $W\left(u_{i},\;v_{i}\right)$ represents geographically weighted (GW) matrix of the selected kernel function that depends on the coordinates $\left(u_{i},v_{i}\right)$ of the $i^{th}$ location. In this study the Bi-square kernel function was used in determining the geographically weighted matrix. The Bi-square kernel function is defined as (Binbin et al. 2014):

(2) $$w_{ij}=\left\{ \begin{array}{ll} {\left[1-\left(d_{ij}/\tau^{2}\right)\right]}^{2}, & if\;d_{ij}\leq \tau \\ \text{0,} & \text{otherwise} \end{array}\right.$$

where $d_{ij}$ represents the distance between locations iandj,andτrepresents the bandwidth that controls the size of the neighborhood. The GWPCA requires prior specification of the optimal bandwidth and number of components to retain (Lu et al. 2014). The number of GWPCs to retain was determined based on the eigen value greater than one approach as used in the conventional PCA. In this study, the bandwidth was obtained automatically using leave-one-out cross-validation approach (Harris et al. 2011). With the cross-validation approach, all possible bandwidths were considered with the optimal bandwidth corresponding to the value with smallest cross-validation score (Collini et al. 2015). The decomposition of the GW variance-covariance matrix provides the local eigen-structure at spatial location $\left(u_{i},v_{i}\right)$ defined as:

(3) $$L\left(u_{i},\;v_{i}\right)V\left(u_{i},\;v_{i}\right)L{\left(u_{i},\;v_{i}\right)}^{\text{T}}=\sum \left(u_{i},\;v_{i}\right)$$

where $L\left(u_{i},\;v_{i}\right)$ represents a matrix of eigenvectors (loadings of each variable on each component) and $V\left(u_{i},\;v_{i}\right)$ represents a diagonal matrix of the eigenvalues (variance of the corresponding principal component). The scores associated with the local component can be defined as:

(4) $$T\left(u_{i},\;v_{i}\right)=\text{XL}\left(u_{i},\;v_{i}\right)$$

The outputs from GWPCA are extensive and provide detailed information which will be obscured in the conventional PCA. For example, a spatial map of the winning variable which corresponds to the variable with the largest absolute loading in each GWPCA can be used to describe the dominant variable in each component and where they are located. The spatial map of the cumulative proportion of total variance (PTV) gives information on where the PTV are highly concentrated. In addition, the spatial map of the GW correlation among the variables can be used to describe the interaction of the variables at distinct spatial locations. Similar to the conventional PCA, the geographically weighted correlation coefficient at location *i* for two given variables $y_{1i\;}\text{and}\;y_{2i}$is computed by:

(5) $$r_{i}=\frac{\mathrm{cov}\left(y_{1i},\;y_{2i}\right)}{s\left(y_{1i}\right)\;s\left(y_{2i}\right)}$$

where $\mathrm{cov}\left(y_{1i},\;y_{2i}\right)$ represents the GW covariance between variables $y_{1i\;}\text{and}\;y_{2i}$at the ._._ location, $s\left(y_{1i}\right)\;\text{and}\;s\left(y_{2i}\right)$ are GW standard deviation for variables $y_{1i\;}\text{and}\;y_{2i}$respectively. The GW covariance is defined as:

(6) $$\mathrm{cov}\left(y_{1i},\;y_{2i}\right)=\frac{\sum_{j=1}^{n}w_{ij}\left[\left(y_{1j}-{\bar{y}}_{1i}\right)\left(y_{2j}-{\bar{y}}_{2i}\right)\right]}{\sum_{j=1}^{n}w_{ij}}$$

where the GW standard deviation and the GW mean are respectively defined as:

(7) $$s\left(y_{i}\right)=\sqrt{\frac{{\sum_{j=1}^{n}w_{ij}\left(y_{j}-{\bar{y}}_{i}\right)}^{2}}{\sum_{j=1}^{n}w_{ij}}}$$

(8) $${\bar{y}}_{i}=\frac{\sum_{j=1}^{n}y_{j}w_{ij}}{\sum_{j=1}^{n}w_{ij}}$$

Prior to the specification of the GWPCA, the conventional PCA was first explored. The analysis was performed on the standardized data in order to control for high variability in some of the heavy metals. In addition, the use of standardized data will prevent the metals with high variance to dominate in the first principal component, thereby making all the heavy metals equally important (Lu et al. 2014; Fernández et al. 2018). The data were standardized using their z-scores. Let $y_{i}$denote a particular soil heavy metal variable of interest, then the z-score for *y*_*i*_ is defined as:

(9) $$z_{i}=\frac{y_{i}-\bar{y}}{s_{y}}$$

where $\bar{y}$ and $s_{y}$represent the mean and standard deviation of the variable, respectively. Thus, the rescaled variable has a mean of zero and a standard deviation of one. All the analysis and computations were conducted using *R* version 4.0.2 (R Core Team, 2020).

## 3 Results and discussion

### 3.1 Conventional principal component analysis

The analysis was conducted on eight soil heavy metals with each consisting of 94 observations. The descriptive statistics of the soil heavy metals concentration (mg/kg) at the sampling points are presented in Table 1. On average, the concentration of Cd was higher in the study area followed by As whilst Cu was the least as indicated by the median of all the variables. The coefficients of skewness for all the soil heavy metals suggest that the distributions of the metals are positively skewed (Table 1). The variation in the soil heavy metal concentration as indicated by the median absolute deviation was higher for Zn than for other soil heavy metals. The correlation matrix shows that the correlations among all the heavy metals are positive and most of them are significantly correlated (Table 2). Whilst some metals are highly correlated (As and Cd; Mn and Ni; Fe and Ni) others are weakly correlated (Zn and Ni; Zn and Mn; Zn and Cr). Thus, the soil heavy metals with significant correlation suggest the possibility of their common origin and transport whilst uncorrelated soil heavy metal suggest the possibility of different origin and transport (Li et al. 2013; Zhang et al. 2019).

**Table 1:** Descriptive statistics of heavy metals concentration (mg/kg) at the sampling locations∗.

Variable	NB	Minimum	Maximum	Mean	LQ	Median	UQ	MAD	Skewness
As	94	1.000	190.980	19.923	4.733	13.105	23.548	13.077	3.964
Cr	94	0.100	10.900	2.468	1.210	2.045	3.315	1.275	1.997
Cu	94	0.570	7.320	1.950	1.223	1.770	2.308	0.801	2.419
Fe	94	0.610	6.140	2.306	1.340	1.990	2.780	1.038	1.216
Mn	94	0.160	13.850	3.122	1.243	2.620	3.893	2.039	1.883
Ni	94	1.000	10.190	2.746	1.565	2.345	3.438	1.416	1.590
Zn	94	0.700	60.510	5.662	1.693	3.195	5.965	2.565	4.495
Cd	94	7.100	209.900	38.175	14.978	31.160	44.970	22.291	2.644

∗ NB, LQ, UQ, and MAD represent the number of observations, lower quartile, upper quartile, and median absolute deviation, respectively.

**Table 2:** Correlation matrix of the eight heavy metals (unstandardised data).

Variable	As	Cr	Cu	Fe	Mn	Ni	Zn	Cd
As	1							
Cr	0.398^a^	1						
Cu	0.325^a^	0.372^a^	1					
Fe	0.386^a^	0.712^a^	0.533^a^	1				
Mn	0.170	0.605^a^	0.378^a^	0.763^a^	1			
Ni	0.334^a^	0.641^a^	0.516^a^	0.814^a^	0.840^a^	1		
Zn	0.128	0.025	0.455^a^	0.064	0.016	0.012	1	
Cd	0.942^a^	0.511^a^	0.518^a^	0.532^a^	0.339^a^	0.479^a^	0.387^a^	1

a Significant at 5% alpha level.

The results of the conventional PCA are presented in Table 3 where the first three PCs have eigenvalues greater than one. These three PCs cumulatively account for 86% of the total variations in the data and were retained for further analysis. The first PC accounts for 53% whilst the second and third PCs individually accounts for less than 20% of the variations in the data. The first component is heavily loaded with Fe whilst the second and third components are heavily loaded with Zn. The three identified PCs may be interpreted as a new uncorrelated variables whose characteristics represent those constitute metals with the largest loadings (Li et al. 2016). However, in the presence of any spatial heterogeneity in the data may mislead the interpretation of the results from the conventional PCA. For instance, it may affect the selection of the most influential variable in each principal component. It may also affect the amount of variation explained by the selected principal components.

**Table 3:** Eigenvalues, proportion of total variance (PTV), cumulative PTV and loadings from the conventional principal component analysis∗.

Variable	PC1	PC2	PC3	PC4	PC5	PC6	PC7	PC8
Eigen values	4.267	1.544	1.046	0.442	0.388	0.187	0.127	0.000
PTV	0.533	0.193	0.131	0.055	0.048	0.023	0.016	0.000
Cumulative PTV	0.533	0.726	0.857	0.912	0.961	0.984	1.000	1.000
Loadings								
As	0.306	0.413	0.555	0.127	0.120	0.037	0.073	0.624
Cr	0.379	-0.179	0.133	-0.591	**-0.628**	0.245	-0.026	0.043
Cu	0.329	0.220	-0.449	**0.599**	-0.461	0.175	0.196	0.025
Fe	**0.427**	-0.220	-0.045	0.020	-0.048	**-0.873**	0.027	0.029
Mn	0.371	-0.387	-0.159	-0.095	0.478	0.280	0.606	0.056
Ni	0.416	-0.288	-0.076	0.195	0.263	0.248	**-0.754**	0.037
Zn	0.118	**0.534**	-**0.600**	-0.477	0.232	-0.067	-0.133	0.195
Cd	0.383	0.427	0.281	-0.028	0.155	0.032	0.040	**-0.751**

∗ The largest absolute loadings are shown in boldface.

### 3.2 Geographically weighted principal component analysis

**Figure 2:**
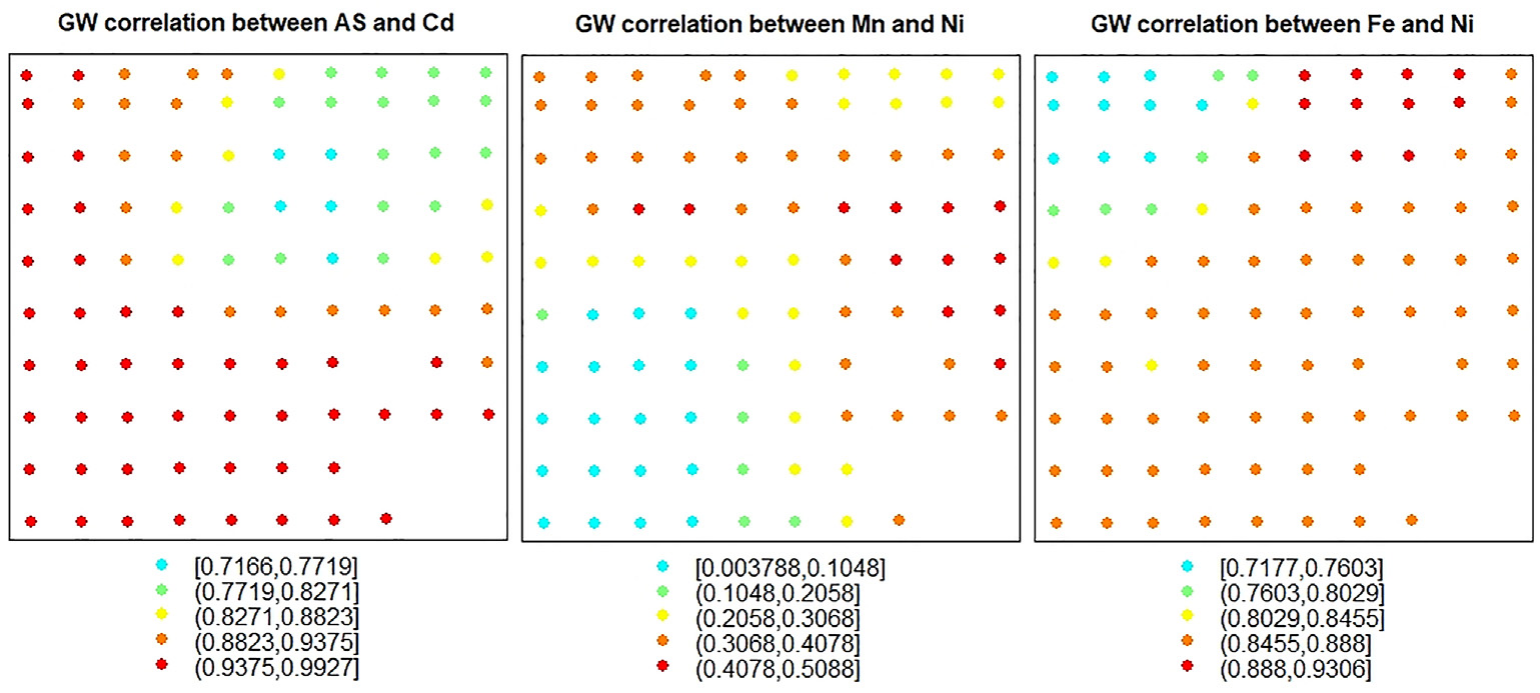
Spatial map of GW correlation between As and Cd (left panel), Mn and Ni (middle panel), Fe and Ni (right panel).

Following, the conventional PCA, the GW correlation was conducted to investigate the local relationship between the first three most correlated variables and determine if such relationship is heterogeneous in the study region. The results, as presented in Figure 2, suggest that the relationship between As and Cd appears heterogeneous, where the relationship is strongest in the southwestern part of the study area and decreases towards the northeastern part. The reverse of this local relationship was observed between Mn and Ni. In the case of Fe and Ni, the relationship was strongest in the southeastern part of the study region and gradually decreased towards the northwestern part. The relationship between other remaining variables also appears heterogeneous (See Supplementary File 1). The presence of heterogeneous local relationships among some of the variables in the study area will undermine the results from the conventional PCA. That is, such local heterogeneity should be taken into account when calculating the principal components.

**Figure 3:**
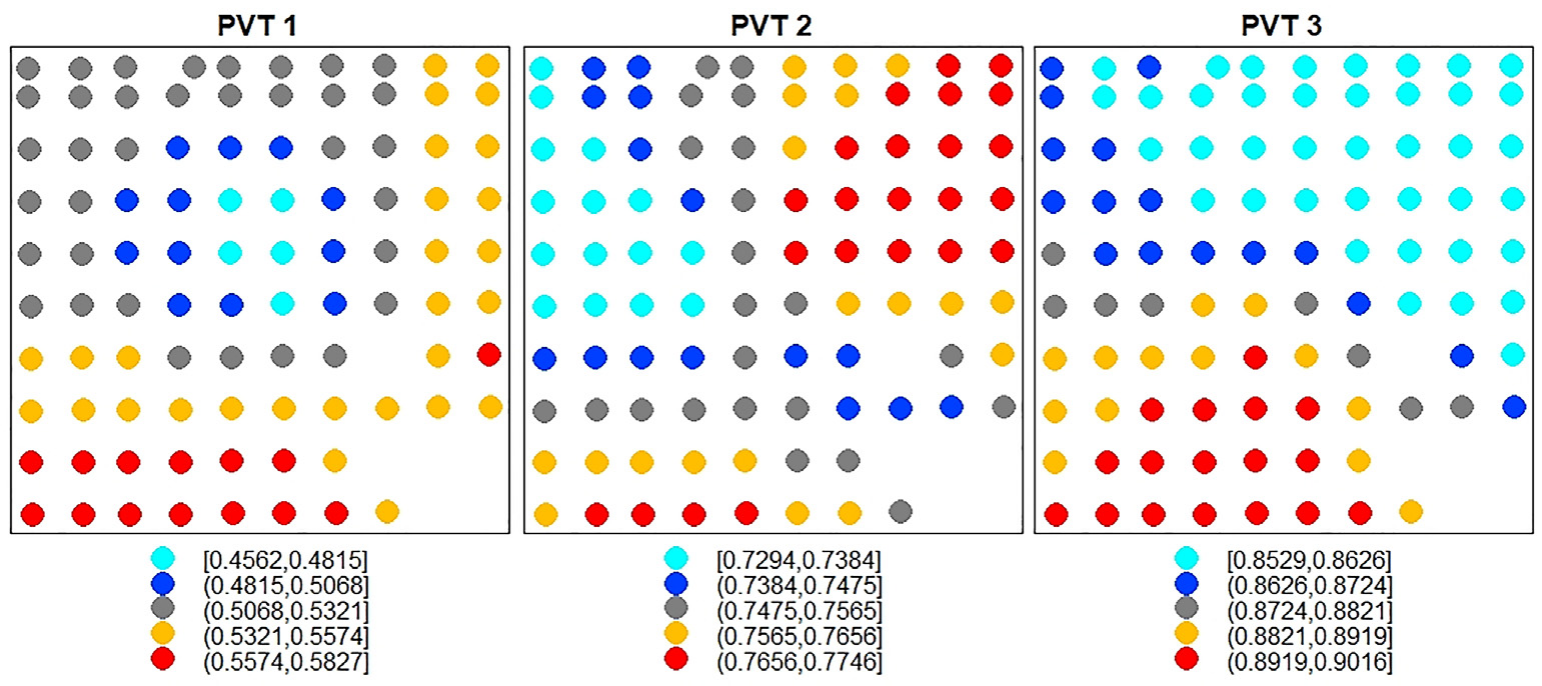
Spatial distribution of the cumulative PTV of the first three geographically weighted principal components.

Since three PCs accounted for 86% of the total variation in the data as observed in the conventional PCA, it was reasonable to retain these same three components for the GWPCA (Li et al. 2016). The spatial maps of the cumulative proportion of total variance (PTV) of the three geographically weighted principal components (GWPCs) show that the PTV is spatially heterogeneous across the study area (Figure 3). It is observed from Figure 3 that the cumulative PTV associated with GWPC 1 is highest at the southern part and least at the central part of the study area. For GWPC 2, high cumulative PTV is concentrated at the northeastern part whilst small values are located at the northwestern part of the study area. In the case of GWPC 3, the cumulative PTV is highest in the southern part and gradually decreases towards the northeastern part of the study area. The cumulative PTV of the three components from the GWPCA is mostly higher (right panel of Figure 3) compared to that of the conventional PCA. The proportion of variance explained by the three GWPC ranged between 85% and 89% (See Supplementary File 1). Thus, in terms of proportion of variance explained, the results of the GWPCA marginally improved on that of conventional PCA.

**Figure 4:**
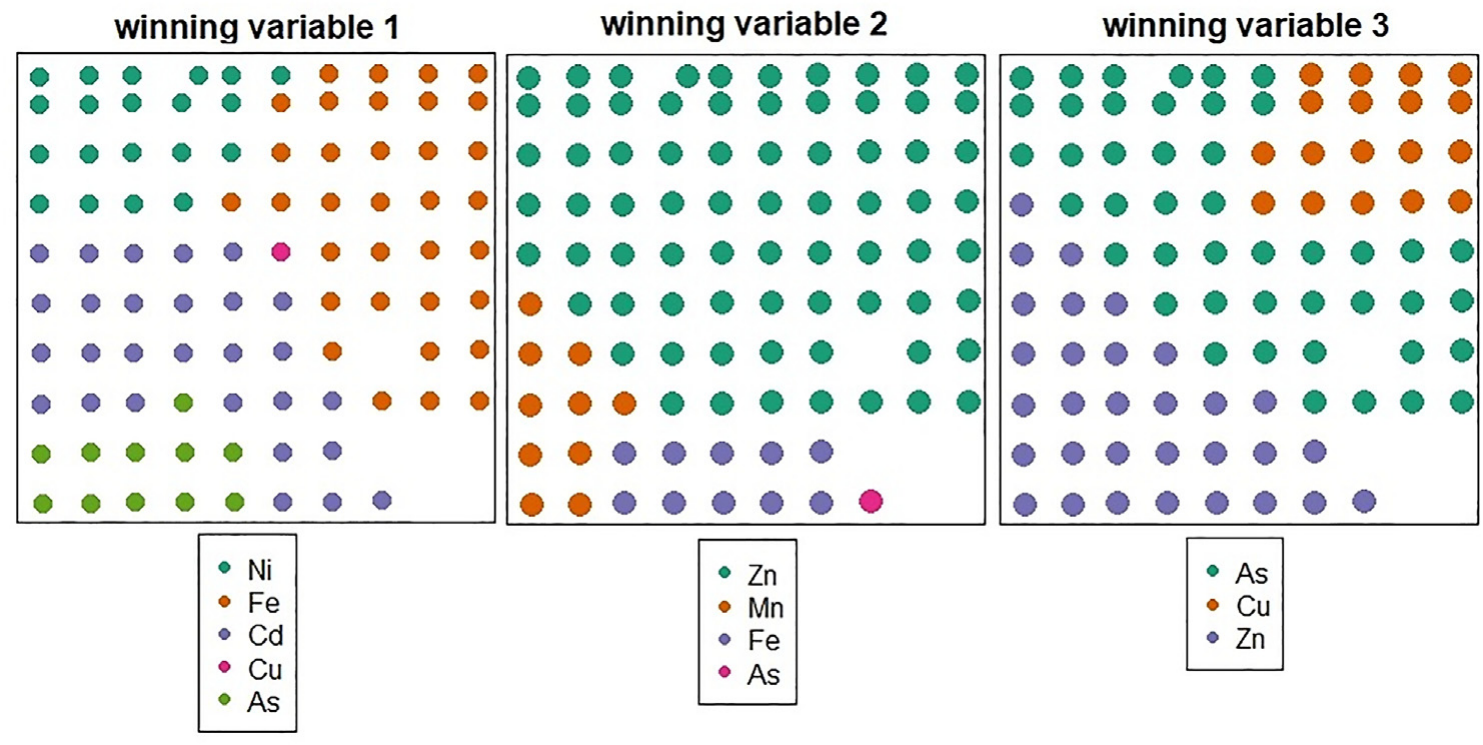
Spatial distribution of the heavy metals with largest loadings on the first three components from GWPCA.

The heavy metals dominating in each GWPC (winning variables) are presented in Figure 4. The figures also describe the spatial distribution of each winning heavy metal for each component. The first GWPC is largely influenced by Fe followed by Cd, Ni and As. For this component, Ni is distributed at the northwestern, Fe is distributed at the northeastern whilst Cd and As are distributed at the southwestern part of the study region. The second GWPC is largely dominated by Zn followed by Fe, Mn, Ni and As. For this component, Zn is distributed at central towards the northern whilst the remaining heavy metals under this component are distributed at the southwestern part of the study region. The third GWPC is largely dominated by As followed by Zn and Cu. For this component, Zn is distributed at the southwestern, As spread from the central to the northeastern whilst Cu is distributed at the northeastern part of the study region. High concentration of Fe, Cd, Zn and As, in the study area may be influenced by anthropogenic sources (Dias and Edwards 2003). The anthropogenic sources of high Fe, Zn and Cu in the northern part of the study area may be linked to the residue of large vehicle repair workshops and spare parts shops located in those areas (Hutton 1983; Iddrisu et al. 2002; Dias and Edwards 2003). The northern part of the study area covers locations such as Suame Magazine where the largest auto mechanic workshops within the West African sub-region is located. In addition, majority of the vehicle spare parts dealers and metal engineering workshops are also found in this area. Residue from the activities of these industries could contribute to the sources of these influential heavy metals in the area. The sources of Zn, As and Cd in the southern part of the study area may be link to transportation related sources (human impact) such as brake pads, wear of tires, and dispersion of lubricants oil, coal, and incineration of industrial and household waste (Yatkin and Bayram 2008; Cannon and Horton 2009; Le Roux et al. 2016). The southern part of the study area covers locations such as Atonsu and Kaasi where most of the local manufacturing industries are surrounded. In addition, majority of the timber industries and the largest abattoir are also found in this area. Examples of heavy metals from these industries includes Cd, As, Zn, and Fe. So the incineration from all these industries could contribute to the sources of these heavy metals in the southern part of the study area.

## 4 Conclusion

The characterization of heavy metal pollution in soil has increasingly become important to understand the sources of pollution and contamination. The present study explored the use of GWPCA as oppose to the conventional PCA to account for spatial heterogeneity and connectivity in soil heavy metals in Kumasi, Ghana. The results from the GWPCA confirm the existence of local relationship among the soil heavy metals in the study area. In addition, the distribution of these variables was found to be spatially heterogeneous in the study area. The identified GWPCs and the spatial distribution of their influential heavy metals can inform the future trajectory of soil pollution and contamination research, allowing for identification of different locations in which these are likely to manifest. The results also have policy implications to alleviate aspect of polluted areas. The use of GWPCA has improved the results of the conventional PCA by providing information on the spatial distribution of the percentage of variance explained in the data and also the distribution of the most influential metals in each identified principal components. Such information gives better understanding of the relationship between the different heavy metals and their distribution across the study area.

## Declarations

### Author contribution statement

Eric N. Aidoo: Conceived and designed the experiment; Performed the experiment; Analyzed and interpreted the data; Wrote the paper.

Simon K. Appiah: Conceived and designed the experiment; Analyzed and interpreted the data.

Gaston E. Awashie: Analyzed and interpreted the data; Wrote the paper.

Alexander Boateng: Conceived and designed the experiment; Wrote the paper.

Godfred Darko: Contributed reagents, materials, analysis tools or data.

### Funding statement

This research did not receive any specific grant from funding agencies in the public, commercial, or not-for-profit sectors.

### Data availability statement

The authors do not have permission to share data.

### Declaration of interests statement

The authors declare no conflict of interest.

### Additional information

No additional information is available for this paper.
